# Application and evaluation of topical amphotericin B for the treatment of respiratory fungal infections

**DOI:** 10.1186/s12879-024-09342-9

**Published:** 2024-04-24

**Authors:** Ning Cui, Jingming Zhao

**Affiliations:** Department of Respiratory and Critical Care Medicine, The Affiliated Hospital of Qingdao University, Qingdao, 266000 Shandong China

**Keywords:** Amphotericin B, Topical administration, Fungal infection

## Abstract

**Background:**

In recent years, the prevalence of respiratory fungal diseases has increased. Polyene antifungal drugs play a pivotal role in the treatment of these conditions, with amphotericin B (AmB) being the most representative drug. This study aimed to evaluate the efficacy and safety of topical administration of AmB in the treatment of respiratory fungal infections.

**Methods:**

We conducted a retrospective study on hospitalized patients treated with topical administered AmB for respiratory fungal infections from January 2014 to June 2023.

**Results:**

Data from 36 patients with invasive pulmonary fungal infections treated with topical administration of AmB were collected and analyzed. Nebulization was administered to 27 patients. After the treatment, 17 patients evidenced improved conditions, whereas 10 patients did not respond and died in the hospital. One patient experienced an irritating cough as an adverse reaction. Seven patients underwent tracheoscopic instillation, and two received intrapleural irrigation; they achieved good clinical therapeutic efficacy without adverse effects.

**Conclusion:**

The combined application of systemic antifungal treatment and topical administration of AmB yielded good therapeutic efficacy and was well-tolerated by the patients. Close monitoring of routine blood tests, liver and kidney function, and levels of electrolytes, troponin, and B-type natriuretic peptide supported this conclusion.

## Background

In recent years, the prevalence of respiratory fungal diseases has increased along with the application of broad-spectrum antibiotics, anti-tumor drugs, immunosuppressants, glucocorticoids, and lung transplantation. Polyene antifungal drugs play a pivotal role in the treatment of these conditions, with amphotericin B (AmB) being the most representative drug. AmB has been used in clinical applications for more than 50 years. It is a polyene antifungal drug derived from the culture medium of *Streptomyces*. AmB binds to the sterols in the fungal membrane, inducing the leakage of intracellular components and fungal cell death. AmB embeds into the double-layer phospholipid structure of the cell membrane, altering the membrane structure, fluidity, and protein function, resulting in fungicidal effects [1]. Most fungi are sensitive to AmB and, in some cases, AmB is the only effective drug for treating critical fungal infections. However, intravenous AmB use is hindered by adverse reactions. Some researchers have found that topical administration can reduce the adverse reactions caused by AmB, complementing the clinical antifungal treatment [2].

In this study, we collected data on patients treated with topical administration of AmB for respiratory fungal infections in our hospital from January 2014 to June 2023. Patients’ data on clinical characteristics, treatment outcomes, and adverse reactions were analyzed, and the scope of application, the application strategy, and the long-term prospects of topical administration of AmB in the treatment of respiratory fungal infections were evaluated.

## Materials and methods

This study included 36 patients with invasive pulmonary fungal infections treated with topical administration of AmB in our hospital from January 2014 to June 2023. The methods of topical administration included nebulization (27 cases), tracheoscopic instillation (7 cases), and intrapleural irrigation (2 cases).

All patients had clinical and imaging signs of pulmonary infection, positive culture and/or pathologic microscopic findings on sputum, alveolar lavage fluid, pleural fluid, etc., and all patients met the international diagnostic criteria for "proven" pulmonary invasive fungal disease [3, 4].

### Treatment strategies

AmB nebulization: nebulization of AmB at a dose 5 ~ 10 mg per administration was performed. The drug was dissolved in sterile water for injection to achieve a final concentration of 0.2 ~ 0.3% before application. The drug was inhaled 2 ~ 3 times per day.

Tracheoscopic instillation: Involved administering AmB at a dose of 5 ~ 25 mg per administration. The drug was dissolved in sterile water for injection to achieve a final concentration of 0.025 ~ 0.125% before application.

Intrapleural irrigation: AmB was administered at a dose of 5 ~ 25 mg per administration. The drug was dissolved in sterile water for injection to achieve a final concentration of 0.01 ~ 0.05% before application.

The main dosage forms of AmB include amphotericin B deoxycholate, amphotericin B lipid complex, liposomal amphotericin B, and others. Among the instructions of the above several drugs, only the instructions of deoxycholate amphotericin B have the usage of topical administration. The formulation for topical administration was deoxycholate amphotericin B in all cases.

### Observation indicators

Patient information, including age, sex, basic illnesses, and department of admission, along with laboratory test results upon admission and after treatment (results of the routine blood tests, results of liver and kidney function tests, and electrolytes levels), treatment strategies (treatment duration, dosage, and the application of combined medications), and adverse reactions and outcomes (mortality rate) were recorded.

## Results

### Basic clinical information

Among the 36 adult patients, 24 were males and 12 were females aged 31–87 (55.7 ± 12.1) years. Six patients were older adults. Twenty-seven cases were treated for *Aspergillus* infection, four for Mucor infection, two for *Rhizopus* infection, one for *Gibberella fujikuroi* infection, one for histoplasmosis, and one for yeast infection. Moreover, 11 patients had malignancies or underwent chemotherapy. Four patients presented with autoimmune diseases, eight were receiving glucocorticoids or immunosuppressive therapy, 14 presented with diabetes mellitus, one presented with chronic obstructive pulmonary disease (COPD), two presented with bronchiectasis, and three presented with pulmonary fibrosis. As for the application of the combined treatment, seven patients received combined intravenous AmB injections, 29 patients received a combined application of voriconazole, and eight patients received a combined application of caspofungin. None of the patients were treated with fluconazole. Among the departments of admission, 19 patients were from the Department of Respiratory Medicine, nine patients were admitted to the intensive care unit (ICU), four patients were from the Department of Transplantation, and four patients were from the Department of Hematology. In this study, 26 patients evidenced improvement in their health conditions after the treatment, whereas 10 patients did not respond to the treatment and died during hospitalization.

A total of 27 patients, including 18 males and nine females aged 31–87 (54.3.5 ± 12.8) years, received nebulization of AmB. Twenty-one of these patients were treated for *Aspergillus* infection, three for Mucor infection, one for *Rhizopus* infection, one for *Gibberella fujikuroi* infection, and one for yeast infection. Eight patients had malignancies or were receiving chemotherapy, three patients had autoimmune diseases, eight patients were receiving glucocorticoids or immunosuppressive therapy, nine patients had diabetes mellitus, one patient had COPD, one patient had bronchiectasis, and three patients had pulmonary fibrosis. As for the application of the combined treatment, four patients received combined intravenous AmB injections, 23 patients received the combined application of voriconazole, and four patients combined application of caspofungin. None of the patients were treated with fluconazole. Among the departments of admission, 10 patients were from the Department of Respiratory Medicine, nine patients were in the ICU, four patients were from the Department of Transplantation, and four patients were admitted to the Department of Hematology. Seventeen patients evidenced improvement in their health condition after the treatment, whereas 10 patients did not respond to the treatment and died during hospitalization. The duration of the nebulizer treatment was 10.2 ± 6.6 days, with the longest duration being 24 days.

Seven patients, five males and two females aged 42–73 (59.9 ± 9.2) years were treated with the tracheoscopic instillation of AmB. Among these seven patients, five were treated for *Aspergillus* infection, one for Mucor infection, and one for *Rhizopus* infection. One patient had malignancy and was receiving chemotherapy, one patient had an autoimmune disease, five patients had diabetes mellitus, and one patient had bronchitis. Combined treatment with intravenous injection of AmB was administered to two patients, whereas combined voriconazole treatment was prescribed to five patients. All seven patients were admitted to the Department of Respiratory Medicine and were discharged after their conditions improved with the treatment. The number of instillations received by these patients varied from 1–6 times, with the highest dose of a single instillation at 25 mg. Four patients underwent left-lung installations, two had right-lung installations, and one underwent installations of both lungs.

Two patients, one male and one female aged 63 and 57 years, respectively, underwent intrapleural irrigation of AmB. One of these patients was treated for *Aspergillus* infection, whereas the other was treated for histoplasmosis. Both patients had comorbid malignancies or underwent chemotherapy. The patient with histoplasmosis received combined treatment with itraconazole, whereas the patient with *Aspergillus* infection received voriconazole treatment. Both patients were admitted to the Department of Respiratory Medicine and were discharged with improved conditions after treatment. The patient with histoplasmosis received left intralpleural irrigation eight times, whereas the patient with *Aspergillus* infection underwent right intrapleural irrigation seven times. The dose of AmB increased gradually from the initial 5 mg to a maximum of 25 mg per irrigation.

### Adverse events

Topical administration of AmB had a good safety profile. Among all patients, only one patient had an adverse reaction of severe cough after nebulization, yielding an adverse reaction rate of 2.8%. No adverse reactions have been found for tracheoscopic instillation and intrapleural irrigation.

### Representative cases

#### A case of treatment with nebulized AmB

**Fig. 1:**
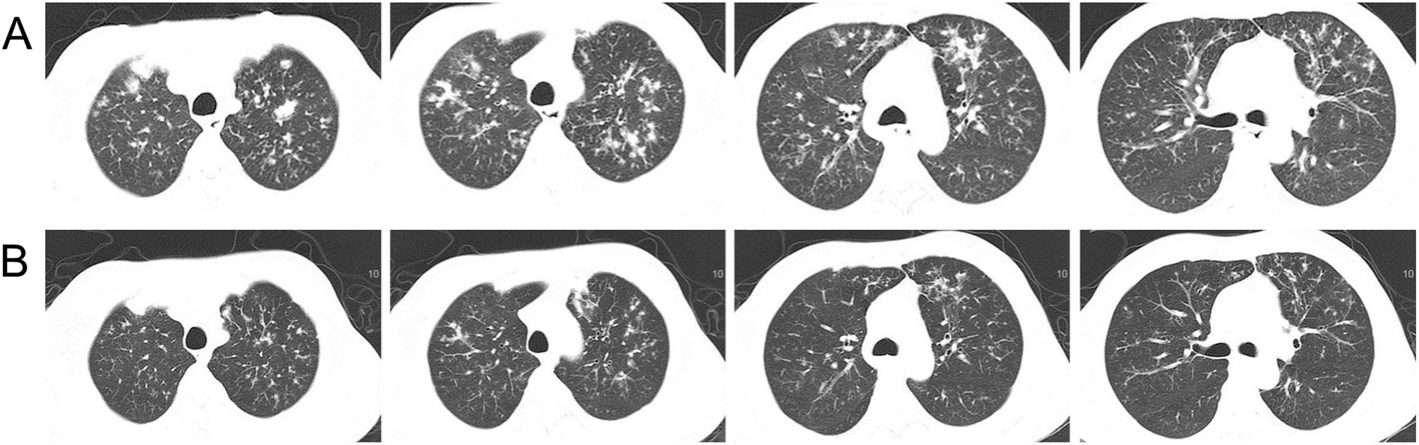
**A** CT scans showed multiple hyperdense shadows in both lungs with bronchiectasis. **B** CT scans evidenced improvements after 1.5 months

A 58-year-old male with comorbid basic illnesses including primary biliary cirrhosis, portal hypertension, splenomegaly, ascites, esophageal and gastric varices, and steroid-induced diabetes, who had been under a long-term treatment with prednisone acetate and ursodeoxycholic acid capsules, was admitted on April 24, 2023, primarily for "intermittent cough and fever for more than 3 months, which was aggravated half a month ago." The patient was infected with SARS-CoV-2 more than 3 months before the admission and presented symptoms such as fever (with the highest body temperature reaching up to 38.5 ˚C), cough, and expectoration. He was diagnosed with pneumonia at the local hospital and was treated with anti-infection medications and anti-inflammatory glucocorticoids. Two weeks before admission to our hospital, the patient had aggravated cough and expectoration, as well as a fever (with the highest body temperature reaching up to 37.9˚C) and blood-tinged sputum. The routine blood test results were as follows: white blood cell count: 18.32*10^9^/L; neutrophil count: 16.68*10^9^/L; whole blood C-reactive protein (CRP) level: 70.46 mg/L; and procalcitonin (PCT) level: 0.763 ng/mL. The results for liver function tests were as follows: albumin level: 28.40 g/L; total bilirubin level: 34.30 µmol/L; alanine transaminase (ALT) level: 83.00 U/L; and aspartate transaminase level: 75.00 U/L. The result of the galactomannan (GM) test was 7.68 S/CO. Computed tomography (CT) scans showed multiple hyperdense shadows in both lungs with bronchiectasis (Fig. 1A). Two sputum cultures suggested *Klebsiella pneumoniae* and *Aspergillus fumigatus* infections. After admission, the patient received anti-fungi treatment including intravenous infusion of 200 mg voriconazole twice per day and of 5 mg nebulized AmB twice per day for 27 days, combined with the anti-bacterial treatment using piperacillin /tazobactam along with the treatment for primary diseases and the comorbidities including diabetes mellitus, gastrointestinal bleeding, ascites, and hypoproteinemia. After the treatment, the patient had his body temperature controlled within the normal range and reduced expectoration. The patient was followed up for 1.5 months after discharge, and his condition was stable. The follow-up chest CT scans evidenced improvements (Fig. 1B).

#### A case of treatment with tracheoscopic AmB instillation

**Fig. 2:**
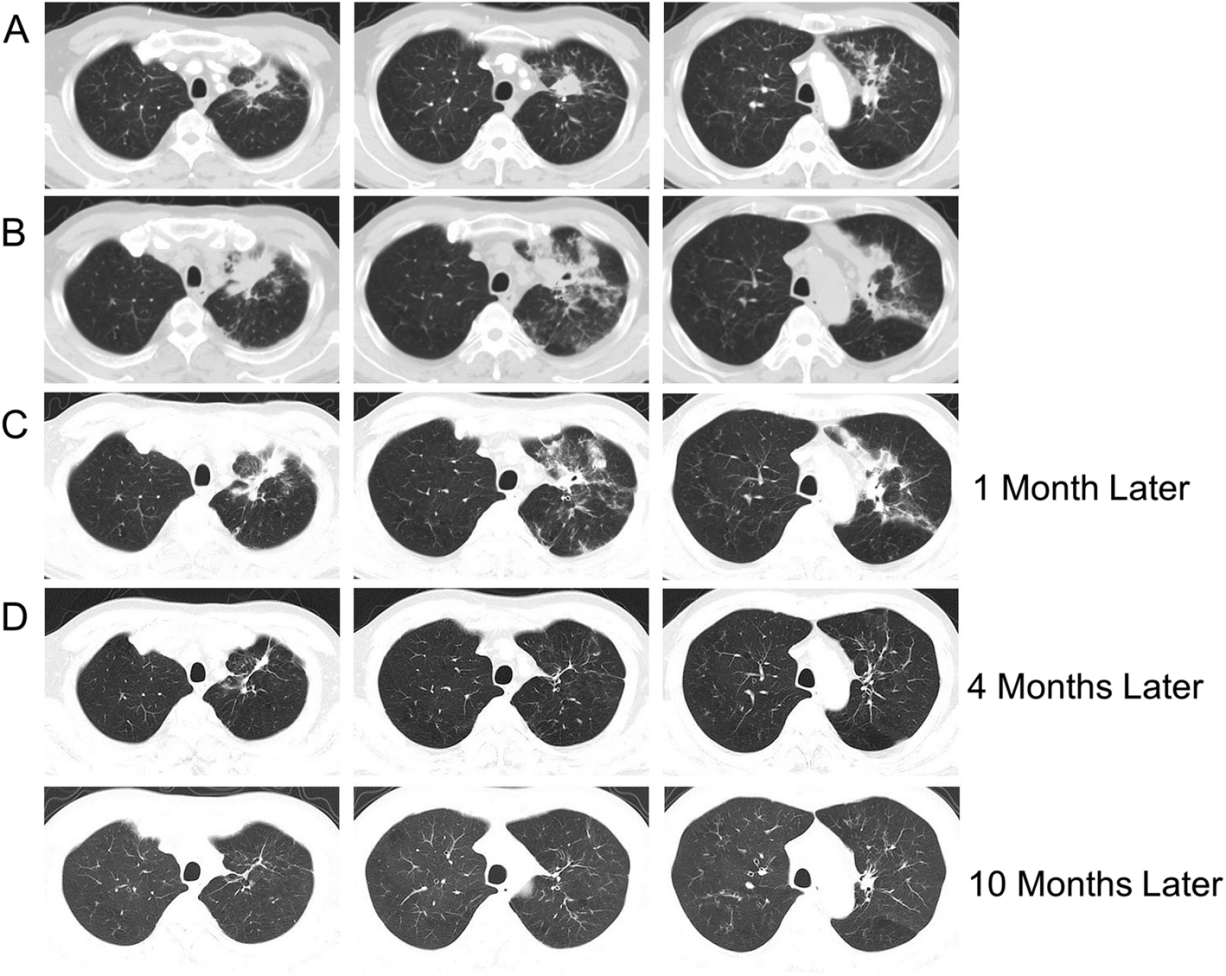
**A** The CT scan suggested a high possibility of inflammation development with abscess formation in the left upper lobe. **B** CT scans showed progress compared to before after voriconazole treatment for 2 months. **C** CT scans evidenced improvements nebulized AmB and tracheoscopic AmB instillation for 1 month. **D** CT scans showed the patient was cured at follow-up

**Fig. 3:**
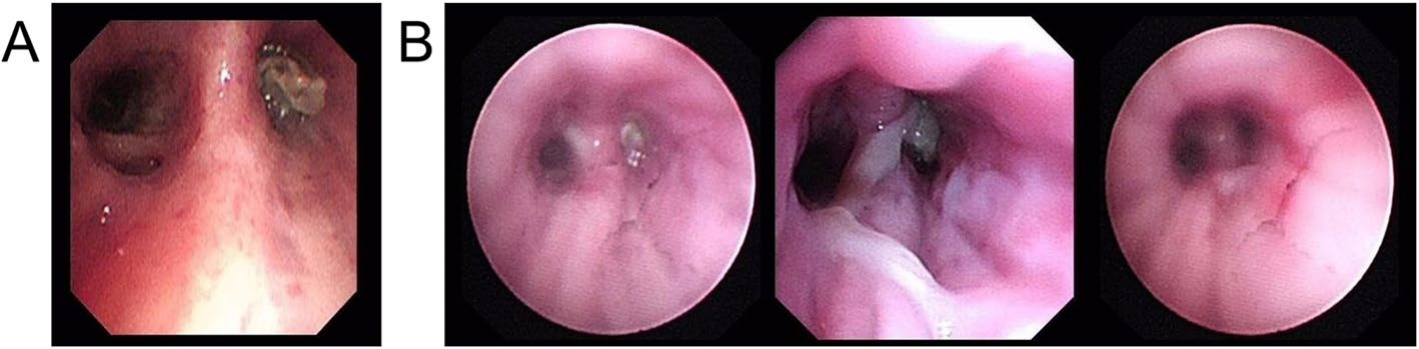
**A** Bronchoscopy showed necrotic tissue at the opening of the left upper lobe bronchus. **B** The necrotic tissue at the opening of the left upper lobe bronchus was gradually reduced and finally disappeared after multiple tracheoscopic AmB instillation

**Fig. 4:**
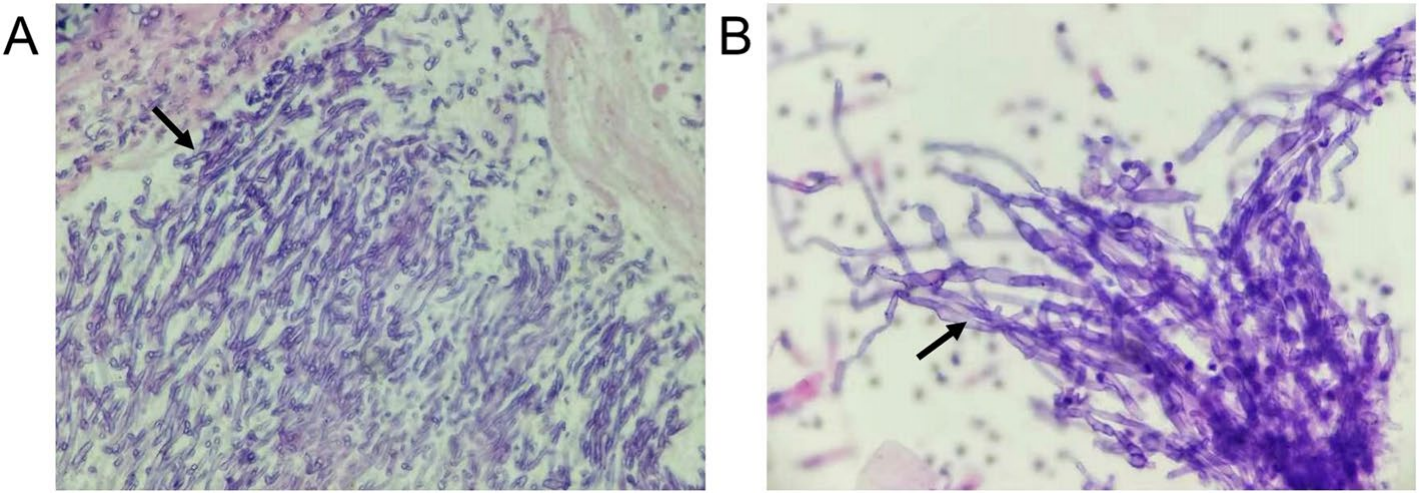
**A** A small amount of fungi with the morphology of Aspergillus was observed in the tissue sample from the biopsy of the left upper lobe. **B** Some loose hyphae with the morphology of Aspergillus were observed in the left upper lobe lavage

A 60-year-old male with histories of stroke, coronary heart disease, and diabetes mellitus, was admitted on January 21, 2021, primarily for “repeated coughing and expectoration for 1 year, which was aggravated 3 days ago." The patient showed symptoms of coughing and expectoration 1 year before admission and was not systemically diagnosed and treated. The results of the routine blood tests 2 months before admission were as follows: white blood cell count: 21.08*10^9^/L; neutrophil count: 17*10^9^/L; monocyte count: 1.51*10^9^/L; CRP level: 161.22 mg/L; and PCT level: 0.248 ng/mL. The CT scan suggested a high possibility of inflammation development with abscess formation in the left upper lobe (Fig. 2A). Bronchoscopy was performed, and necrotic tissue was observed at the opening of the left upper lobe bronchus (Fig. 3A). The result of the GM test on the lavage was > 5.00 μg/l. The pathological examinations showed that the tissue sample from the biopsy of the left upper lobe had chronic suppurative inflammation with the formation of abscess and fibrous granulation tissue. A small amount of fungi with the morphology of *Aspergillus* was observed. Some loose hyphae with the morphology of *Aspergillus* and moderate neutrophil infiltration were both observed in the left upper lobe lavage (Fig. 4). In the beginning, the patient was treated with intravenous administration followed by oral administration of voriconazole. The patient's symptoms were not relieved, and the CT scans at the follow-up showed progress compared to before (Fig. 2B). Subsequently, 5 mg nebulized AmB twice per day was additionally administered for 3 days, followed by the administration of 10 mg nebulized AmB twice per day for 10 days. Bronchoscopy was performed multiple times for the removal of the necrotic tissue in the left upper lobe and the instillation of diluted AmB into the anterior segment of the left upper lobe bronchus (10 mg of AmB was diluted in a 30 mL solution and was administrated twice through the sheath; 25 mg of AmB was diluted to a 25 mL solution and was administrated 3 times through the sheath). After the treatment, the patient had relieved symptoms and improved CT scan results and was discharged (Fig. 2C). The observations of bronchoscopy also suggested improvements in the patient’s condition (Fig. 3B). Oral administration of voriconazole was continued at discharge and the patient was cured at follow-up (Fig. 2D).

#### A case of treatment with intrapleural AmB irrigation

**Fig. 5:**
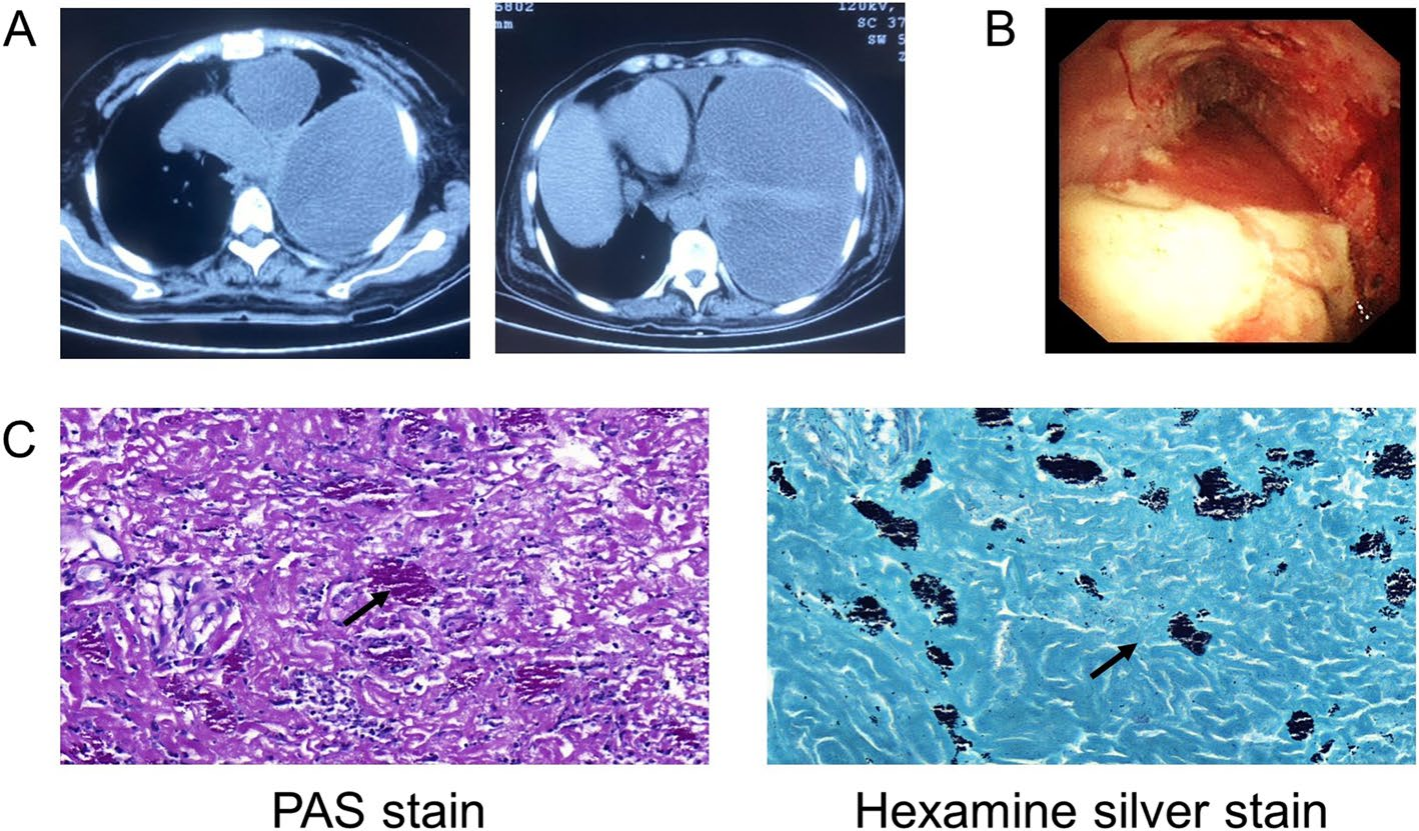
**A** CT showed left pleural effusion. **B** The medical thoracoscopy revealed extensive purulent attachmented to the parietal pleura, visceral pleura and diaphragmatic pleura. **C** PAS staining showed spore capsules in pink (black arrows). Silver staining showed spore capsules in black (black arrows)

**Fig. 6:**
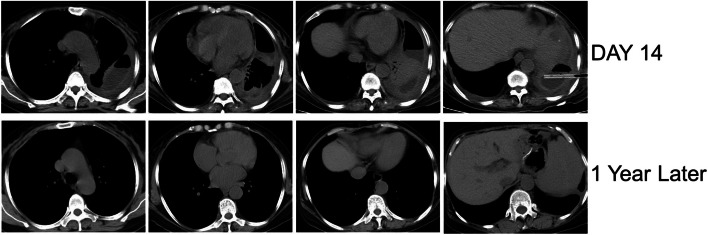
Show the chest CT scans of the patient on the 14th day of hospitalization and 1 year later, showing the left lung was gradually re-expanded, the left pleural effusion was gradually reduced, and the left pleural thickening was gradually decreased

A 57-year-old woman with gastric cancer and pre-existing diabetes mellitus had a massive left-sided pleural effusion suggested by a chest CT scan (Fig. 5A). The culture of the pleural fluid suggested *Escherichia coli* and Streptococcus *constellatus* infections. Medical thoracoscopy was performed after admission. Extensive purulent attachments to the parietal pleura, visceral pleura, and diaphragmatic pleura were observed (Fig. 5B). Histopathology of the thoracoscopic pleural biopsy after hexamine silver and PAS staining supported *Histoplasma* infection (Fig. 5C). *Histoplasma* infection was treated with intravenous injection of AmB at an initial dose of 3 mg and intrapleural irrigation of AmB at an initial dose of 5 mg. Both intravenous injection and intrapleural irrigation of amphotericin B were gradually increased by 5 mg per day to 25 mg per day for maintenance dosing. Intravenous injection infusion was administered for 15 days, and intrapleural thoracic irrigation infusion was administered for 8 days. The patient’s pleural effusion and necrotic tissue in the pleural cavity were significantly reduced in a short time, and the clinical symptoms were significantly improved. After discharge, the patient recovered well and had no evident complications or sequelae (Fig. 6).

## Discussion

Pulmonary fungal infections, especially *Aspergillus* infections, have the following characteristics: rapid dissemination, requiring timely intervention with drugs, rapid formation of necrotic tissue and destruction of local lung structure, and rapid encapsulation with fibrous and granulomatous tissue when effectively intervened by the application of antifungal drugs or confined by the host’s immune responses. These characteristics determined that it may be difficult for the interventions with intravenously administrated drugs to exert effect because of poor local blood supply. When AmB is applied intravenously, at the same time point, the drug concentration in pleural fluid is usually less than half the blood concentration, and the drug concentration in bronchial secretions is even lower. Therefore, the drug concentration achieved in the lungs at commonly used therapeutic doses can only have inhibitory effects on the growth of fungi. The drug has good solubility in water and is absorbed slowly in a small amount through the airway mucosa and pleura without causing evident irritations. Based on these pharmacological and metabolic characteristics, topical administration of AmB via nebulization, tracheoscopic instillation or intrapleural irrigation has irreplaceable advantages and is worthy of clinical promotion [5, 6] . Therefore, patients who have little reaction to local treatment and high tolerance can combine topical amphotericin B to improve the efficacy and shorten the course of treatment.

### Nebulization

Most pathogens that cause pulmonary fungal infections are taken into the lungs by inhalation through the airway, making it important to increase local drug concentrations in the lung tissue for treatment. Nebulization of AmB allows the drug to reach the lesion rapidly at a high local concentration. With this administration method, the drug concentrations in saliva and sputum are similar to the plasma concentrations, which not only exerts a local antifungal effect but also reduces the adverse effects caused by the interactions between systemically administerd drugs [7, 8]. The results of several studies suggest that nebulized AmB can be retained in alveolar epithelial lining fluid and lung tissues at a proper concentration for longer while the blood drug concentration remains low, avoiding the systemic toxicity associated with high blood concentration [9]. Therefore, the drug concentration can be maintained at a high level in the lungs for a long time after nebulized AmB, while the blood drug concentration remains low, which helps avoid the systemic toxicity induced by the high drug concentration in the blood. There were no significant differences in the therapeutic efficacies between AmB with different forms. To reduce the adverse effects of nebulization, the concentration of inhaled drugs should be limited, the speed of drug intake via inhalation should be initially slow and gradually increased, and the volume of drugs to be nebulized should be initially small and gradually increased to allow patients to slowly adapt during the process. The most common adverse reactions to the nebulization of AmB are cough, dysgeusia, and nausea [6, 10].

Multiple studies suggest that the nebulization of AmB in combination with systemic antifungal drugs is significantly more effective than using nebulized AmB alone in the treatment of systemic fungal infections in animals [11]. Most cases included in this study received combined intravenous or oral administrations of azole antifungal drugs and treatment with nebulized AmB at a dose range of 5 ~ 25 mg per administration, 2 ~ 3 times per day. During the treatment period, only one case presented an adverse reaction of an irritating cough that was relieved after stopping the drug use. The rest of the patients did not present symptoms of irritation, suggesting a good tolerance for the treatment. The cough symptoms were generally relieved in most of the patients after the treatment, and bronchoscopy during the follow-up visits evidenced that the local lesions were significantly resorbed. The GM test for the bronchoalveolar lavage fluid had a negative result, and the lung lesions identified by medical imaging were gradually resorbed in imaging. No fungal growth was observed in the cultures of sputum or the lavage fluid. The patients had stable symptoms and vital signs at the follow-up after discharge, and there was no further deterioration of the status of blood cells and liver or kidney function.

The nebulized case we selected had typical imaging of pulmonary fungal infection with dissemination along the bronchial tree. Therefore, we added nebulized AmB to the systemic antifungal therapy. In addition to efficacy and tolerability, our treatment allowed to obtain significant economic savings and to maintain a good adherence to treatment by the patient.

### Tracheoscopic instillation

When systemic application of antifungal drugs is ineffective or not tolerated and causes adverse events, the instillation of antifungal drugs directly into the intrapleural lesions has achieved promising clinical results in treating the lesions of fungal infections [12, 13]. Bronchoscopic bronchoalveolar lavage has become an important technique in the treatment of severe lung infections. Compared with traditional treatments, bronchoalveolar lavage flushes out the mucous secretions obstructing the bronchi, delivers drugs directly to the infected lung tissues, and ensures that the topical drug concentration is in the range that can effectively kill the pathogens [14]. During topical administration of drugs through the bronchoscopic sheath, efforts should be made to avoid delivering drugs into uninfected bronchial and lung tissues besides the target lesions. The application of 3-dimensional (3D) reconstructed CT scans and virtual or electromagnetic navigation bronchoscopy can perfectly solve this problem [2, 15].

This study included seven patients who underwent tracheoscopic irrigation. The lesions in the lungs were precisely located and the entry route of the bronchoscope was carefully determined with the assistance of the 3D reconstructed chest CT scan. A special sheath was inserted deeply into the distal end of the target bronchus for instilling the drugs. By referring to the methods described in previous studies [16], the patient's position was adjusted to avoid direct leakage of the drugs into the bronchial tree (the patient should stay in this position postoperatively for as long as possible to retain the drugs at the site of the lesion to the fullest extent). The seven patients under observation had improved conditions and no chills, fever, rash, nausea, vomiting, hypokalemia, liver and kidney function impairment, or any common side effects caused by the intravenous administration of AmB.

In the tracheoscopic instillation case we selected, obvious necrotic tissue was observed under tracheoscopy and a small amount of fungi with the morphology of Aspergillus was observed by pathological examination. Targeted tracheoscopic instillation of AmB into the lumen of the trachea could increase the local drug concentration, with a gradual reduction of necrotic tissue on tracheoscopy, enhancing the efficacy and shortening the treatment course.

### Intrapleural irrigation

Fungal infections of the pleural space are relatively rare in clinical practice; however, their rapid progression and high severity endanger the patients and hinder their recovery. Therapeutic concentrations of antifungal drugs can hardly be achieved in the pleural cavity with conventional intravenous administration methods. Intravenous AmB achieves a higher cumulative amount in the body over a longer time. Intravenous AmB should be started in small doses and takes 5–7 days to reach the dose required at the therapeutic level. The low local concentration of antifungal drugs is the primary reason for the unsuccessful treatment of fungal empyema. Successful treatment of fungal empyema requires maintaining the concentration of the drug in the pleural cavity above its minimum inhibitory concentration (MIC). The thickness of the pleura makes it difficult for AmB to diffuse into the pleural effusion, and the drug concentration in the pleural cavity is low [17–20]. The concentration of AmB in the patient's pleural fluid was lower than the MIC, making it unlikely to exert an effective antifungal effect. Guazzelli et al. reported in 2012 that AmB lavage treatment achieved good results in three cases of fungal empyema [21]. In recent years, researchers adapted the strategy of topical administration of antifungal drugs by intrapleural lavage and achieved satisfactory results [21, 22] . In this study, we collected data from two cases treated with intrapleural irrigation of AmB for fungal empyema, with one case of infection with Aspergillus empyema and the other of Histoplasma-associated empyema. Both cases had good treatment outcomes. However, the application of AmB for pleural lavage is rare in clinical practices, which requires the researchers to further summarize the treatment experience and standardize the treatment procedures.

Intrapleural irrigation is essential for thoracic infections. In our selected case of thoracic infection caused by Histoplasma, we observed good efficacy of local intrapleural AmB irrigation combined with systemic therapy, with no systemic or local adverse effects.

For antifungal therapy, effective and inexpensive new drugs with minor adverse effects need to urgently be developed. It is necessary to adapt the techniques of topical drug administration to improve the therapeutic efficacy and reduce the adverse effects associated with the systemic administration of existing drugs. However, it is crucial to recognize the adverse effects that changes in the method of administration may carry. In conclusion, there is a lack of validation by large-scale evidence-based medical studies for the topical administration of antifungal drugs to treat respiratory infections. Currently, treatment with topical drug administration is still the main adjuvant therapy for patients who respond poorly or are intolerant to intravenous therapy. Most reports on successful cases are reports of individual patients. This paper is an observational study in which all cases were treated by topical AmB in combination with systemic therapy, and there was no control group for systemic therapy only. This is a limitation of the study. In the future, controls can be set up to better observe the efficacy and side effects of topical amphotericin B treatment. Furthermore, a large number of clinical trials are needed to verify the efficacy and safety of this treatment strategy. In clinical practice, the decision regarding using topical drug administration measures based on the specific conditions of the patients can be made under the premise of following the principles of antifungal treatment. Meanwhile, patients’ reactions to drugs should be closely monitored.

## Data Availability

All data generated or analyzed during this study are included in this published article.

## Abbreviations

ALT: Alanine transaminase
AmB: Amphotericin B
COPD: Chronic obstructive pulmonary disease
CRP: C-reactive protein
CT: Computed tomography
GM: Galactomannan
ICU: Intensive care unit
MIC: Minimum inhibitory concentration
PCT: Procalcitonin
3D: 3-Dimensional

## References

[1] Brajtburg J, Powderly WG, Kobayashi GS, Medoff G (1990). Amphotericin B: current understanding of mechanisms of action. Antimicrob Agents Chemother.

[2] Kuiper L, Ruijgrok EJ (2009). A review on the clinical use of inhaled amphotericin B. J Aerosol Med Pulm Drug Deliv.

[3] Donnelly JP, Chen SC, Kauffman CA (2020). Revision and update of the consensus definitions of invasive fungal disease from the European Organization for Research and Treatment of Cancer and the Mycoses Study Group Education and Research Consortium. Clin Infect Dis.

[4] Patterson TF, Thompson GR, Denning DW (2016). Practice guidelines for the diagnosis and management of aspergillosis: 2016 Update by the Infectious Diseases Society of America. Clin Infect Dis.

[5] Gavaldà J, Martín MT, López P (2005). Efficacy of nebulized liposomal amphotericin B in treatment of experimental pulmonary aspergillosis. Antimicrob Agents Chemother.

[6] Rijnders BJ, Cornelissen JJ, Slobbe L (2008). Aerosolized liposomal amphotericin B for the prevention of invasive pulmonary aspergillosis during prolonged neutropenia: a randomized, placebo-controlled trial. Clin Infect Dis.

[7] Shah SP, Misra A (2004). Liposomal amphotericin B dry powder inhaler: effect of fines on in vitro performance. Pharmazie.

[8] Hullard-Pulstinger A, Holler E, Hahn J, Andreesen R, Krause SW (2011). Prophylactic application of nebulized liposomal amphotericin B in hematologic patients with neutropenia. Onkologie.

[9] Husain S, Capitano B, Corcoran T (2010). Intrapulmonary disposition of amphotericin B after aerosolized delivery of amphotericin B lipid complex (Abelcet; ABLC) in lung transplant recipients. Transplantation.

[10] Borro JM, Solé A, de la Torre M (2008). Efficiency and safety of inhaled amphotericin B lipid complex (Abelcet) in the prophylaxis of invasive fungal infections following lung transplantation. Transplant Proc.

[11] Takazono T, Izumikawa K, Mihara T (2009). Efficacy of combination antifungal therapy with intraperitoneally administered micafungin and aerosolized liposomal amphotericin B against murine invasive pulmonary aspergillosis. Antimicrob Agents Chemother.

[12] Denning DW, Cadranel J, Beigelman-Aubry C (2016). Chronic pulmonary aspergillosis: rationale and clinical guidelines for diagnosis and management. Eur Respir J.

[13] Kravitz JN, Berry MW, Schabel SI, Judson MA (2013). A modern series of percutaneous intracavitary instillation of amphotericin B for the treatment of severe hemoptysis from pulmonary aspergilloma. Chest.

[14] Nattusamy L, Kalai U, Hadda V, Mohan A, Guleria R, Madan K (2017). Bronchoscopic instillation of liposomal amphotericin B in management of nonresponding endobronchial mucormycosis. Lung India.

[15] Usuda J (2018). Virtual Bronchoscopic Navigation( VBN) and Electromagnetic Navigation System. Kyobu Geka.

[16] Yamada H, Kohno S, Koga H, Maesaki S, Kaku M (1993). Topical treatment of pulmonary aspergilloma by antifungals. Relationship between duration of the disease and efficacy of therapy. Chest..

[17] Weiler S, Bellmann-Weiler R, Joannidis M, Bellmann R (2007). Penetration of amphotericin B lipid formulations into pleural effusion. Antimicrob Agents Chemother.

[18] Kutty K, Neicheril JC (1987). Treatment of pleural blastomycosis: penetration of amphotericin B into the pleural fluid. J Infect Dis.

[19] Moriyama B, Torabi-Parizi P, Pratt AK (2010). Pharmacokinetics of liposomal amphotericin B in pleural fluid. Antimicrob Agents Chemother.

[20] Moriyama B, Ditullio M, Wilson E (2011). Pharmacokinetics of anidulafungin in pleural fluid during the treatment of a patient with Candida empyema. Antimicrob Agents Chemother.

[21] Guazzelli LS, Severo CB, Hoff LS, Pinto GL, Camargo JJ, Severo LC (2012). Aspergillus fumigatus fungus ball in the pleural cavity. J Bras Pneumol..

[22] Bonatti H, Lass-Floerl C, Angerer K (2010). Successful management of postpneumonectomy Aspergillus pleural empyema by combined surgical and anti-fungal treatment with voriconazole and caspofungin. Mycoses.

